# A Novel G542X CFTR Rat Model of Cystic Fibrosis Is Sensitive to Nonsense Mediated Decay

**DOI:** 10.3389/fphys.2020.611294

**Published:** 2020-12-16

**Authors:** Jyoti Sharma, Joseph Abbott, Lauren Klaskala, Guojun Zhao, Susan E. Birket, Steven M. Rowe

**Affiliations:** ^1^Department of Medicine, University of Alabama at Birmingham, Birmingham, AL, United States; ^2^Gregory Fleming James Cystic Fibrosis Research Center, University of Alabama at Birmingham, Birmingham, AL, United States; ^3^Horizon Discovery Group, PLC, St. Louis, MO, United States

**Keywords:** cystic fibrosis, nonsense mutation G542X, rat model, rat epithelial cells, translational readthrough

## Abstract

Nonsense mutations that lead to the insertion of a premature termination codon (PTC) in the cystic fibrosis transmembrane conductance regulator (CFTR) transcript affect 11% of patients with cystic fibrosis (CF) worldwide and are associated with severe disease phenotype. While CF rat models have contributed significantly to our understanding of CF disease pathogenesis, there are currently no rat models available for studying CF nonsense mutations. Here we created and characterized the first homozygous CF rat model that bears the CFTR G542X nonsense mutation in the endogenous locus using CRISPR/Cas9 gene editing. In addition to displaying severe CF manifestations and developmental defects such as reduced growth, abnormal tooth enamel, and intestinal obstruction, CFTR G542X knockin rats demonstrated an absence of CFTR function in tracheal and intestinal sections as assessed by nasal potential difference and transepithelial short-circuit current measurements. Reduced CFTR mRNA levels in the model further suggested sensitivity to nonsense-mediated decay, a pathway elicited by the presence of PTCs that degrades the PTC-bearing transcripts and thus further diminishes the level of CFTR protein. Although functional restoration of CFTR was observed in G542X rat tracheal epithelial cells in response to single readthrough agent therapy, therapeutic efficacy was not observed in G542X knockin rats *in vivo*. The G542X rat model provides an invaluable tool for the identification and *in vivo* validation of potential therapies for CFTR nonsense mutations.

## Introduction

Cystic fibrosis (CF) is an autosomal recessive disease that affects 1 in 2,500 births among the people of European descent (Rowe et al., 2005; Quon and Rowe, 2016). As a multi-organ disease, CF primarily affects epithelial cells in the intestine, respiratory system, pancreas, gall bladder, and sweat glands (Quon and Rowe, 2016; De Boeck et al., 2017). Approximately 2,000 disease variants have been described in the *CFTR* gene, and many lead to a disease phenotype (Mutyam et al., 2016; Guimbellot et al., 2017). Of these, premature termination codon (PTC) mutations affect 11% of CF patients worldwide.

The substitution of single base pair in the genome leads to the insertion of a premature stop codon (UGA, UAG, or UAA) in the open reading frame of messenger RNA (mRNA), which is also subject to degradation by the cellular surveillance mechanism nonsense-mediated decay (NMD; Chang et al., 2007; Linde et al., 2007; Keeling et al., 2014). People with nonsense mutations exhibit a severe CF phenotype as a result of often severely reduced transcript levels and production of little or no truncated, and mostly non-functional, protein (Wilschanski, 2012). While CFTR potentiators such as ivacaftor (VX-770) or correctors such as lumacaftor (VX-809), tezacaftor (VX-661), or elexacaftor (VX-445) are available for many CFTR mutations (Van Goor et al., 2009; Ramsey et al., 2011; Ren et al., 2013; Wainwright et al., 2015; Guimbellot et al., 2017; Davies et al., 2018; Sala and Jain, 2018), there are no therapies currently available that specifically address CFTR nonsense mutations. Certain drugs, primarily aminoglycosides and ataluren (PTC124), have been shown to induce readthrough at PTCs by facilitating an insertion of near-cognate aminoacyl tRNA at the stop codon site in experimental models (Wilschanski et al., 2003; Malik et al., 2010; Sermet-Gaudelus et al., 2010; McDonald et al., 2017; Sharma et al., 2020). However, none of these compounds has significantly improved clinical outcomes, principally due to insufficient efficacy (Smith et al., 1986; Molitoris, 1997; Linde et al., 2007; Peabody Lever et al., 2020), lack of specificity, or toxicity (Mingeot-Leclercq and Tulkens, 1999; Saleh et al., 2016).

Animal models of CFTR nonsense mutations are essential tools to understand the biological consequences of stop codon readthrough therapy. Current animal models are primarily murine species that have been generated either with no CFTR channel (Clarke et al., 1992; Snouwaert et al., 1992), endogenous knockin (i.e., within the native locus) CFTR G542X mutations (McHugh et al., 2018), or a hypermorph with non-endogenous/transgenic CFTR containing G542X mutations (Du et al., 2002, 2006; Keiser and Engelhardt, 2011; Wilke et al., 2011). Furthermore, CFTR transgenic mouse models express human CFTR cDNA which are not subject to NMD, thus limiting the detection of definite readthrough in *in vivo* settings. Although these mice models have proven useful for understanding the CF intestinal phenotype, they have failed to recapitulate human airway physiology (Scholte et al., 2004), unlike pigs, ferrets, and rats. Therefore, an animal model beyond murine species that exhibits more defined CF lung pathophysiology and expresses CFTR nonsense mutations in an endogenous CFTR locus is needed.

Previously, we developed a CF knockout rat model that enables the longitudinal study of muco-obstructive lung disease characteristic of CF patients (Guilbault et al., 2007; Rogers et al., 2008; Birket et al., 2018), providing some advantages over other available species. More recently, the G551D gating mutation has been introduced into CF rats, permitting pharmacological research (Birket et al., 2020). For the present study, we generated a knockin CF rat expressing the CFTR G542X mutation within its native locus. We also assessed the effects of readthrough treatment on CFTR function *in vivo* in the model, as well as *in vitro* using rat tracheal epithelial cells (RTECs) (Stasi et al., 2015).

## Materials and Methods

### Ethical Approval

This study was carried out in compliance with the guide for the Care and Use of Laboratory Animals of the National Institutes of Health. Protocols were approved by Horizon Discovery, Inc., or University of Alabama at Birmingham (UAB) Institutional Animal Care and Use Committee (IACUC; UAB Approval Number 09479). All procedures were performed under sodium pentobarbital or ketamine/xylazine/acepromazine anesthesia, with all efforts made to minimize animal suffering.

### Generation of the G542X-CFTR Rat Model

**FIGURE 1:**
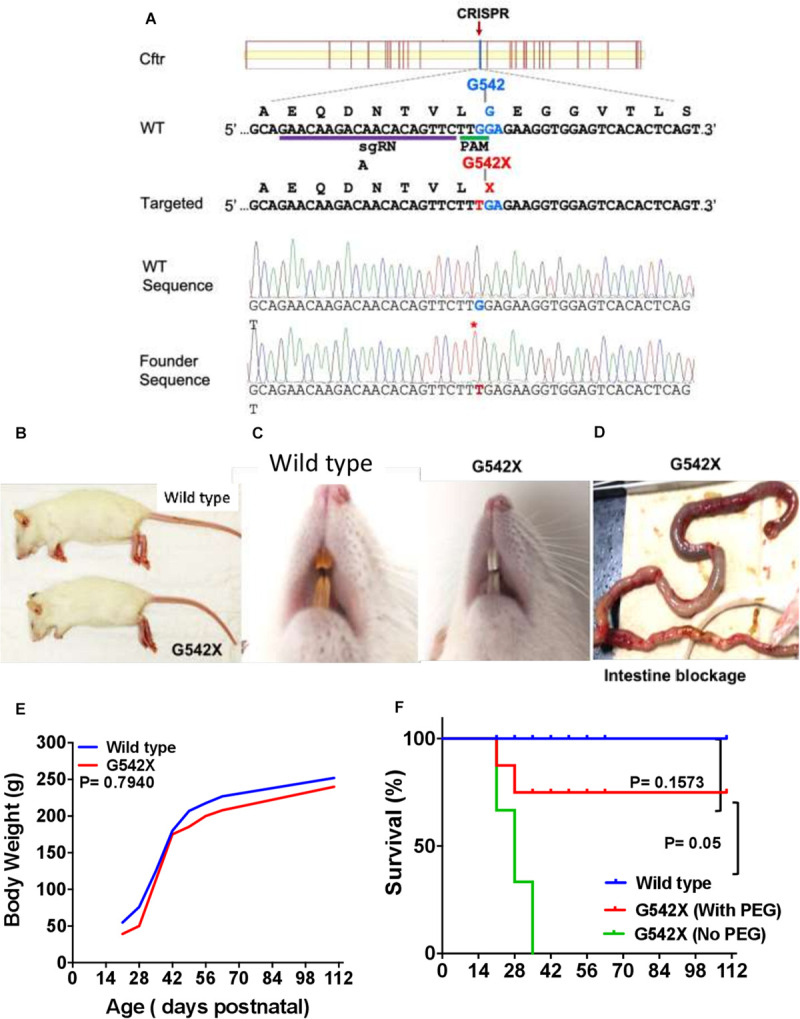
Generation of CF G542X rats. **(A)** A Cas9 sgRNA was designed to target close to the desired Cftr Gly542 encoding site. The partial wildtype (WT) and targeted allele DNA sequence (5′–3′) are listed along with their respective encoding amino acid sequences (N-C, shown above their codons). The gRNA binding site and PAM site (TGG) is underlined. The single point mutation (G to T) mutation changes the amino acid from Gly542 to a stop codon X, both of which are shown in red. The bottom two panels are Sanger sequencing data from a wildtype and a biallelic G542X mutation rats, respectively. **(B)** Body size. **(C)** Dentition shows yellowish brown enamel in wild type **(left)** and white incisors in G542X **(right)**. **(D)** Gross ileal section showing intestinal blockage in G542X rat. **(E)** Mean values of body weight by age from wild type and G542X rats. **(F)** Survival curve for G542X rats with and without treatment with enteral PEG as compared to wild type rats. *N* = 8–10 animals per group.

The *Cftr G542X* point mutation rat model was designed and generated by Horizon Discovery (now Envigo RMS, Saint Louis, MO, United States) using CRISPR-based technology. Specifically, a Cas9 single guide RNA (sgRNA) targeting to the rat *Cftr* gene Gly542 (c.1652)-encoding site was transcribed *in vitro* using T7 RNA polymerase-based *in vitro* transcription methods from a DNA template (Kouranova et al., 2016). The DNA template was amplified by overlapping PCR with a forward oligo DNA containing the T7 promoter, an sgRNA target site, and a reverse oligo DNA complementary to a Cas9 tracrRNA sequence (Kouranova et al., 2016). To validate sgRNA activity, the *in vitro* transcribed sgRNA was purified, quantified, and nucleofected into rat C6 cells that stably express SpCas9 using Lonza’s nucleofection kit. Twenty-four hours post nucleofection, the sgRNA-transfected cells were collected and genomic DNA was extracted. A DNA fragment flanking the sgRNA target site in the rat *Cftr* gene was PCR amplified using Cel1-F and Cel1-R primer pair (Supplementary Table 1). The Cas9/sgRNA cutting efficiency was quantified by Surveyor mutation assay (Transgenomic SURVEYOR kit) using previously described methods (Carbery et al., 2010). The validated active sgRNA targeting GAACAAGACAACACAGTTCT(TGG) of the rat *Cftr* gene was complexed with SpCas9 protein to form ribonucleoprotein (RNP) complex prior to delivery into rat embryos (Figure 1). An oligo donor DNA comprising the G542X coding sequence and ∼68nt homology arms on each side was synthesized by IDT (Supplementary Table 1). The oligo donor DNA along with the Cas9/sgRNA RNP complex was nucleofected into fertilized one-cell stage rat embryos isolated from superovulated Sprague-Dawley donor females using a method described previously (Kouranova et al., 2016). Following microinjection, 25–30 eggs were transferred into each pseudopregnant female rat. Cftr mutant rats were birthed 3 weeks later. Tail or toe biopsies from those live births were collected for genomic DNA extraction and analysis to identify founder rats by PCR and Sanger sequencing. Specifically, genomic DNA extracts from those rats were screened for correct integration of this G542X point mutation by PCR and Sanger sequencing with the Cel1-F and Cel1-R primer pair (Supplementary Table 1). Sanger sequencing data show that out of 42 rats genotyped, at least 15 rats had the expected G542X mutation (representative wild type and G542X rat sequences are shown in the Supplementary Material). Among those 15 founder rats, in addition to the desired G542X mutation, most of those founders also have other NHEJ-based insertion or deletion mutations at the target site, indicating mosaicism genotype in the founder stage. Three samples show a single chromatogram peak at the desired G542X mutation site, suggesting the same biallelic G542X mutation in those three founders (Figure 1). Selected G542X founder rats were backcrossed to wild type Sprague Dawley rats to generate heterozygous F1 rats. The genotype of all F1 rats was revalidated by PCR and Sanger sequencing using the same Cel1 primer set. Animals were bred and housed in standard cages maintained on a 12 h light/dark cycle with *ad libitum* access to food and water. Routine health monitoring of the colony was performed at IDEXX (Columbia, MO, United States) and indicated no evidence of infection with known serious pathogens. All animal generation work at Horizon Discovery was performed in accordance with the approved animal protocol (Protocol # 001) overseen by Horizon Discovery’s IACUC.

### Propagation of the Strain

Male and female Sprague-Dawley CFTR rats heterozygous for the G542X mutation were bred to produce wild type and G542X-homozygous rats. Litters remained with lactating dams through 21 days post birth. Following identification by sequencing, wild type and G542X rats were weaned and were provided regular chow, DietGel^®^ 76A, and water containing polyethylglycol (236 g, GoLytely, Braintree Laboratories, Inc.) to reduce intestinal blockage.

### Gene Sequencing

Tail snips were taken between 10 and 14 days after birth for collection of genomic DNA using the Accuris 1 Hour Mammalian Genotyping Kit (Stellar 87 Scientific, Baltimore, MD, United States). One microliter DNA was mixed with 0.5 μL of each primer (forward 5′TTAACCAGCTAAGTGAATTGCAT and reverse 5′CCCTAGAGACAGAGCACAAGC, Integrated DNA Technologies, Skokie, IL, United States) and evaluated under standard PCR conditions: 95°C for 5 min, followed by 35 cycles of 95°C for 30 s, 60°C for 30 s, and 68°C for 40 s, with a final fixed cycle at 68°C for 5 min, resulting in an amplification of a 357bp sequence spanning the point mutation. Samples were subsequently purified with the USB PCR Product Pre-Sequencing Kit (Thermo Fisher Scientific, Waltham, MA, United States) and sequenced using the Sanger method.

### Western Blotting

Intestinal tissue was homogenized in PBS on ice followed by lysis in RIPA buffer (Thermo Fisher Scientific, Rockford, IL, United States) with Halt protease inhibitor cocktail (Thermo Fisher Scientific), as previously described (Tuggle et al., 2014). Protein quantification was performed using the BCA assay kit (Thermo Fisher Scientific). Samples were mixed with 4× sample buffer and incubated at 37°C for 10 min. Equal amounts of protein (20 μg) were loaded for electrophoresis. Wild type Sprague-Dawley rat trachea and lung extract were used as positive controls for CFTR detection. Membranes were then blocked in 5% non-fat dry milk dissolved in PBST, followed by incubation with monoclonal anti-CFTR (1:3,000; UNC 596) and mouse monoclonal anti-β-actin (1:5,000; Thermo Fisher Scientific), and subsequently goat anti-mouse secondary antibody conjugated to HRP (1:10,000; Dako North America, Inc.). Images were captured by ChemiDocXRS (Bio-Rad) using SuperSignal West Femto ECL kit (Thermo Fisher Scientific).

### Real Time Reverse-Transcriptase Quantitative PCR

RNA was isolated from rat tissues (lung and ileum) and tracheal epithelial cells using an RNAeasy isolation kit (Qiagen). RNA quality was measured using a NanoDrop (Thermo Fisher Scientific). Real-time polymerase chain reaction (RT-PCR) was performed using TaqMan RNA to Ct 1-Step kit in Quant Studio3 applied BioSystems (Thermo Fisher Scientific). Relative transcript levels were normalized to Gapdh. Two additional housekeeping genes (Hprt and Rps9) were also tested in a confirmatory study (Supplementary Figure 2). Primers were purchased from Life Technology (see Supplementary Table 2 for the list of primer ID numbers).

### Nasal Potential Difference

Calomel electrodes and electronic data capture (AD Instruments) were used to measure potential difference as previously described for rats, mice, and humans (Pyle et al., 2010; Solomon et al., 2010; Tuggle et al., 2014; Kaza et al., 2017). Ketamine (200 mg/kg) and xylazine (30 mg/kg) intraperitoneal injection were used to anesthetize rats. Nasal cavities were perfused sequentially with (1) Ringer’s solution containing 140 mM NaCl, 5 mM KCl, 1 mM MgCl_2_, 2 mM CaCl_2_, 10 mM HEPES, and 100 μM amiloride (pH 7.3); (2) with amiloride; (3) Cl^–^free Ringer’s solution (6 mM Cl^–^, pH 7) with amiloride; (4) Cl^–^free Ringer’s solution, amiloride, and forskolin (20 μM); and (5) Cl^–^free Ringer’s solution, amiloride, glybenclamide, and CFTR_Inh_-172 (10 μM) as performed earlier (Tuggle et al., 2014). Each condition was perfused at a steady flow rate of 1.5 ml/h for 5 min or until a stable signal was achieved.

### Intestinal Short-Circuit Current (I_sc_) Measurements

I_sc_ measurements of intestinal ileal sections were obtained using Ussing chamber analysis under voltage clamp conditions as previously described (Du et al., 2002; Tuggle et al., 2014). Four to six tissue segments approximately 5–6 mm in length sectioned 5 cm above the cecum were dissected as previously described (Tuggle et al., 2014), and incubated in TTX (Tetrodotoxin, 3.3 × 10^–4^ μM in PBS) for 10 min to block neuronal action potential by binding to voltage-gated sodium channels. Intestinal segments were mounted as flat sheets onto sliders (area ∼0.16 cm^2^). Bath solutions were constantly circulated with 95% O_2_:5% CO_2_. Ringer’s solution (in mM) 120 NaCl, 25 NaHCO_3_, 3.33 KH_2_PO_4_, 0.83 K_2_HPO_4_, 1.2 CaCl_2_, 1.2 MgCl_2_, and 10 glucose was used for monitoring I_sc_. Ion transport associated with phospholipase C or A2 activity was blocked by adding indomethacin (10 μM) to both chambers. Tissues were equilibrated for 10 min in Ringer’s solution followed by 10 min of recording. Low Cl^–^ Ringer’s contained (in mM) 1.2 NaCl, 25 NaHCO_3_, 3.33 KH_2_PO_4_, 0.83 K_2_HPO_4_, 1.2 CaCl_2_, 1.2 MgCl_2_, 141 Na gluconate, and 10.8 mannitol. Mucosal side chambers were changed to 1:1 regular Ringer’s:low Cl^–^ Ringer’s. After 15–20 min of incubation, forskolin (10 μM) and IBMX (3-Isobutyl-1-methylxanthine, 100 μM) were added to both chambers for 15–20 min, followed by addition of glybenclamide (200 μM) to block forskolin-activated CFTR I_sc_. Because polyethylene glycol (PEG) used to prevent intestinal obstruction can reduce intestinal ion channel activity, PEG was omitted 2-3 days before necropsy.

### Tracheal I_sc_ Measurements

I_sc_ measurements were performed as described in previous studies under voltage clamp conditions in Ussing chambers (Physiologic Instruments) (Tuggle et al., 2014). Briefly, trachea were excised, opened longitudinally, and sectioned into 1–2 segments. Tracheal tissues were mounted as flat sheets in Ussing chambers (area ∼0.031 cm^2^). Chambers were constantly maintained at 37°C and bubbled vigorously with 95% O_2_:5% CO_2_. Tissue segments were equilibrated for 10 min in regular Ringer solution that contained (in mM) 120 NaCl, 25 NaHCO_3_, 3.33 KH_2_PO_4_, 0.83 K_2_HPO_4_, 1.2 CaCl_2_, 1.2 MgCl_2_, and 10 mannitol to establish a baseline. This was followed by administration of CFTR_Inh_-172 (10 μM) to block constitutively active CFTR-dependent chloride current, and then sequential addition of amiloride (100 μM), ATP (10 μM), and bumetanide (100 μM). I_sc_ divergence was calculated after subsequent attainment of stable plateau after baseline and CFTR inhibitor treatment for several minutes for each sample as previously reported (Tuggle et al., 2014).

### Rat Tracheal Epithelial Cell Culture

Rat trachea were dissected and placed immediately in 10 ml RTEC media [500 ml F12 media, 5 ml of 5,000 units/ml Penicillin-Streptomycin (Thermo Fisher Scientific), and 25 μg amphotericin (Sigma Aldrich)]. Once the connective tissue was removed from the trachea, it was processed with lumen exposed in 10 ml Pronase solution [10 ml RTEC media and 15 mg Pronase (Sigma Aldrich)] overnight at 4°C. On day 2, tubes containing tracheal tissue were rocked a few times and kept at 4°C for 30–60 min. Tracheae were then washed a few times using RTEC media (500 ml F 12 media, 100 ml heat inactivated FBS, 25 μg amphotericin) in 15 ml tubes by inverting. RTEC media supernatants were then mixed with the Pronase solution and centrifuged at 1400 rpm for 10 min at 4°C. The pellet was resuspended in 2 ml DNase solution (Sigma Aldrich) [18 ml F12 media, 2 ml BSA (10 mg/ml), and 10 mg DNase1]. The cell suspension was centrifuged at 1400 rpm for 5 minutes at 4°C and the resulting pellet was resuspended in 8 ml of RTEC media (475.5 ml of DMEM/F12 media, 7.5 ml 1M HEPES, 10 ml of 200 mM glutamine, 2 ml of 7.5% NaHCO_3_, 5 ml of 100X Penicillin-Streptomycin, and 25 μg amphotericin). The cell suspension was then plated on a T25 flask and incubated at 37°C for 5 h. Cells were pooled from the flask, and the flask was rinsed twice with RTEC media, followed by centrifugation of the cell suspension at 1,400 rpm for 10 minutes at 4°C. The pellet was resuspended in RTEC media with 5 ml of heat-inactivated FBS and cells were seeded (7.5 × 10^4^–1 × 10^5^ cells/well) on filters pre-coated with collagen-1 at a liquid–liquid interface. Cells were cultured in complete PneumaCult-Ex (Stem Cell Tech) media for one week. After the seventh day, cells were maintained at air–liquid interface with PneumaCult Maintenance (Stem Cell Tech) media supplemented to the basolateral surface only.

### RTEC I_sc_ Measurements

Rat tracheal epithelial cell I_sc_ was assessed similarly as above in Ussing chambers with a baseline and amiloride inhibition measurements in Ringer’s solution followed by addition of low-Cl^–^ + amiloride solution in the mucosal side of the chamber, administration of forskolin (10 μM), and finally CFTR_Inh_-172 (10 μM). I_sc_ alterations resulting from CFTR agonist and antagonist treatments were obtained once a stable plateau was achieved in all electrophysiological measurements.

### Pharmacokinetic Drug Levels by Liquid Chromatography-Mass Spectroscopy

Rats were treated with amikacin (170 mg/kg, Sigma) once daily for 5 days by subcutaneous dosing. Blood samples were collected 24 h before the final dosing from tail veins and 1 h after the final dosing by heart puncture to measure steady state plasma levels using liquid chromatography–mass spectroscopy (LC-MS).

### Statistical Analysis

Data are presented as mean ± SEM or as individual data points. Unpaired two-tailed Student’s *t*-test or one-way ANOVA was used where appropriate for measuring statistical significance. For survival analysis, Kaplan–Meier survival curves were plotted. Analyses were performed using Prism software (GraphPad Inc.), and differences were regarded as statistically significant at *P* ≤ 0.05.

## Results

### Generation of CFTR G542X Knockin Rats and Litter Demographics

G542X knockin rats were generated using CRISPR-Cas targeting of c.1624 to engineer a G > T point mutation (Figure 1A) into fertilized embryos. Guide RNAs were designed to avoid silent mutations and preserve the applicability of this model for testing readthrough agents. In the first 50 microinjections, 15 founders were generated and identified by sequencing as described above. Of the 15 founders, 3 pups were identified to have the targeted point mutation on one allele, without evidence of other variants introduced to the CFTR gene on the second allele. These pups were bred to generate F1. Rats with each genotype were born at the expected Mendelian frequency. Heterozygotes did not display any differences from wild type animals in general appearance and growth.

### CFTR G542X Knockin Rats Exhibit Developmental Defects

CFTR G542X rats displayed development-associated consequences similar to those seen in CFTR knockout rats (Tuggle et al., 2014; Stalvey et al., 2017). Marked growth retardation in homozygous rats was observed after 21 days of age, although body sizes between groups appeared similar at birth. At day 28 after birth, the G542X rats looked smaller in size (Figure 1B), their teeth had thick enamel deposits (Figure 1C), and they exhibited intestinal obstruction typically occurring at the time of weaning (Figure 1D). Although body weight was generally preserved (Figure 1E), intestinal obstruction limited survival (30% survival at 3 weeks after weaning) as compared to wild type rats (100% survival, Figure 1F, *P* < 0.001). The addition of PEG as a dietary supplement improved life expectancy to 70% (*P* = 0.1573 Figure 1F). Together, CFTR-G542X knockin rats exhibited developmental abnormalities, retarded growth, and intestinal obstruction as observed in established CFTR knockout rats (Tuggle et al., 2014) and human subjects (Ferrazzano et al., 2012; Mentessidou et al., 2018).

### Bioelectric Measurements

**FIGURE 2:**
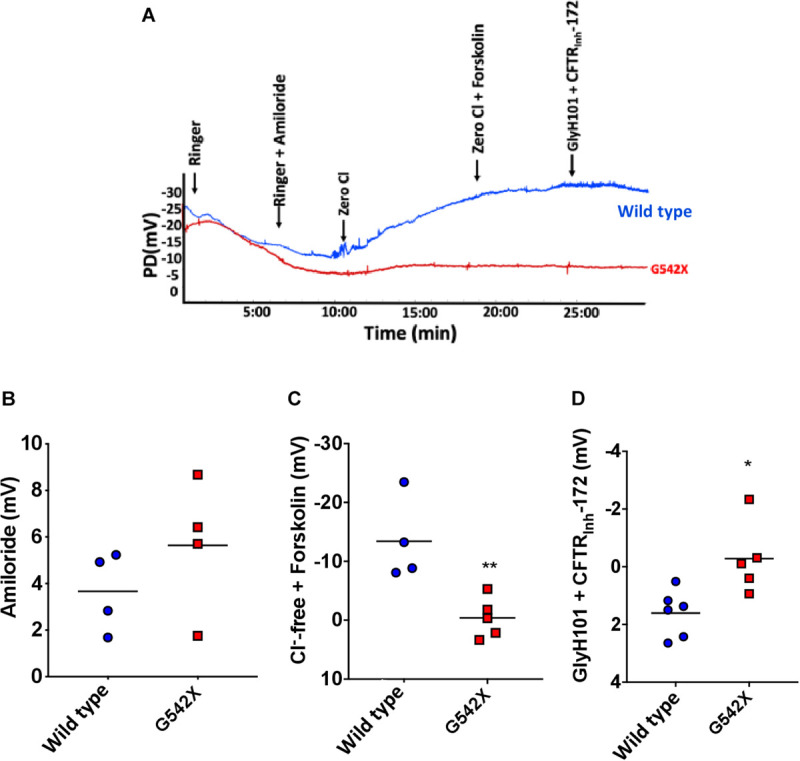
Electrophysiologic defect by nasal potential difference (NPD) in G542X CFTR rats. **(A)** Representative NPD tracings from G542X and wild type rats showing serial perfusion of Ringer’s, Ringer’s + amiloride, zero Cl, zero Cl + forskolin, and Glyh101 + CFTR_Inh_-172. **(B–D)** Summary of change in PD following perfusion with **(B)** amiloride (100 μM), **(C)** Cl^–^ free + forskolin (20 μM), and **(D)** Cl ^–^ free + CFTR inhibitors (GlyH101 + CFTR_Inh_-172, 10 μM each). *N* = 4–6 animals/group. ^∗^*P* < 0.05, ^∗∗^*P* < 0.01 by ANOVA with Tukey *post hoc* testing.

Assessment of nasal potential difference (NPD) has been widely used to evaluate ion transport defects *in vivo* in rat, mouse, and ferret models of CF and other diseases characterized by alterations in ion channel activity (Kaza et al., 2017). We used NPD to assess CFTR activity in the upper airways of G542X and wild type rats (representative tracings, Figure 2A). There was no difference between wild type and G542X rats following perfusion with amiloride (G542X −5.6 ± 1.4 mV, wild type −3.7 ± 0.9 mV, Figure 2B), as we have observed with CF knockout rats (Tuggle et al., 2014; McCormick et al., 2018). G542X rats exhibited no CFTR-dependent hyperpolarization following perfusion with chloride-free Ringer’s solution plus forskolin, whereas responses in wild type littermates were typical of other non-CF species (G542X = −0.4 ± 1.5 mV,wild type = −13.4 ± 3.5 mV, *P* < 0.01, Figures 2A,C) and prior publications (Kaza et al., 2017). Similarly, changes in potential difference after perfusion with CFTR antagonists GlyH101 plus CFTR_inh_-172 showed moderate inhibition in wild type rats, but there was no response in G542X rats (G542X = −0.3 ± 0.6 mV, wild type = 1.6 ± 0.3 mV, *P* < 0.05, Figure 2D). These measurements demonstrate the absence of CFTR activity in the nasal lumen of CFTR G542X rats.

### Tracheal I_sc_ Measurements

**FIGURE 3:**
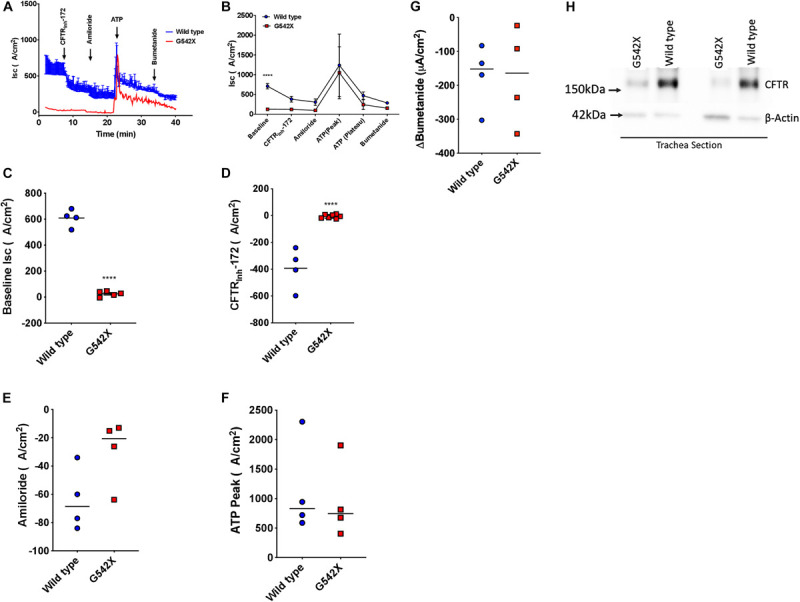
Functional and biochemical defects in the lung of G542X CFTR rats. **(A)** Representative tracheal I_sc_ tracing from G542X and wild type rats with perfusion of CFTR_Inh_-172 (10 μM), amiloride (100 μM), ATP (10 μM), and bumetanide (100 μM). **(B)** Summary of tracheal I_sc_ from G542X and wild type rats. **(C–G)** Summary data showing baseline I_sc_ **(C)**, CFTR_Inh_-172-mediated inhibition **(D)**, amiloride inhibition **(E)**, ATP peak **(F)**, and bumetanide inhibition **(G)** in G542X and wild type rats. Each point represents an individual animal. **(H)** Western blot of CFTR and ß-actin loading control in two pairs of G542X and wild type tracheal sections. *N* = 4–5 animals/group ^∗∗^*P* < 0.01, ^****^P < 0.0001 by ANOVA with Tukey *post hoc* testing.

We next investigated CFTR-dependent ion transport in tracheal tissues evaluated *ex vivo* by I_sc_ analysis, a definitive measure of CFTR activity. Trachea from G542X animals demonstrated diminished baseline I_sc_ compared to wild type (G542X = 25.5 ± 9.1 μA/cm^2^, wild type = 608.8 ± 33.4 μA/cm^2^, *P* < 0.0001, Figures 3A–C), with minimal CFTR_inh_-172-sensitive I_sc_ in G542X relative to wild type (G542X = −5.4 ± 5.5 μA/cm^2^, wild type = −392.7 ± 76.3 μA/cm^2^, *P* < 0.0001, Figures 3A,B,D). The contribution of sodium-dependent currents was comparable for both genotypes, as measured by addition of the ENaC inhibitor amiloride (Figures 3A,B,E), findings consistent with NPD (Figures 2A,B). ATP addition generated non-CFTR dependent currents as evidenced by an acute increase in I_sc_ in both G542X (805.7 ± 236 μA/cm^2^) and wild type rats (945.3 ± 363 μA/cm^2^, Figures 3A,B,F, *P* = NS). Similarly, subsequent bumetanide strongly inhibited ATP-sensitive currents in both wild type and G542X rats (Figures 3A,B,G). In separate studies, we altered the order of ion transport modulators, and applied amiloride and forskolin, followed by ATP and bumetanide in Ussing chamber conditions. In these studies, aside from the difference in baseline currents that recapitulated prior experiments, we observed a compensatory increase of ATP-dependent Cl^–^ transport with truncated CFTR (G542X rats) compared to wild type (Supplementary Figure 1, stimulatory protocol), which has also been seen in CFTR knockout rats and CF patients (Tuggle et al., 2014). Western blot further corroborated functional data, as G542X rats showed diminished CFTR protein expression in tracheal sections compared to wild type CFTR rats (Figure 3H). These findings suggest the absence of CFTR expression and activity in G542X rat airways.

### Intestinal I_sc_ Measurements

**FIGURE 4:**
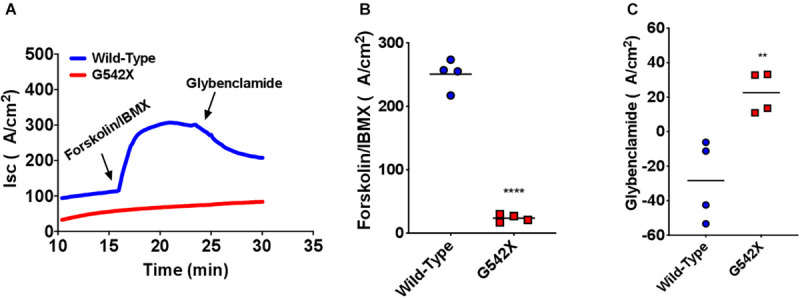
Functional CFTR decrements by I_sc_ analysis of small intestine of G542X rats. **(A)** Representative I_sc_ tracings of ileal sections with serial perfusion of forskolin (10 μM)/IBMX (100 μM) and the inhibitor glybenclamide (200 μM). **(B)** Summary of forskolin/IBMX-stimulated currents. **(C)** Summary of glybenclamide-inhibitable currents. Each point represents an individual animal. *N* = 4 rats/group. ^∗∗^*P* < 0.01, ^****^P < 0.0001 by ANOVA with Tukey *post hoc* testing.

CFTR protein is abundantly expressed in intestinal and rectal tissues (Crawford et al., 1991; Mutolo et al., 2018). To confirm the multi-organ ion transport defect in this rat model, we next examined intestinal Cl^–^ transport in excised ileal segments. *Ex vivo* ileal I_sc_ measurements showed significantly reduced forskolin-stimulated current in G542X rats compared to wild type (G542X = 29.8 ± 4.0 μA/cm^2^, wild type = 188.0 ± 11.7 μA/cm^2^, *P* < 0.0001, Figures 4A,B). This was accompanied by lack of GlyH101 inhibition in G542X rats versus the reduced currents observed in wild type rats (G542X = 29.05 ± 2.77 μA/cm^2^, wild type = −20.59 ± 4.692 μA/cm^2^, *P* < 0.01 Figures 4A,C). These findings indicate the functional absence of CFTR activity in the intestinal ileal epithelium, noting CF rats do have a small amount of non-CFTR dependent forskolin-activated I_sc_.

### Diminished CFTR mRNA Expression in CFTR G542X Knockin Rats Compared to Wild Type Rats

**FIGURE 5:**
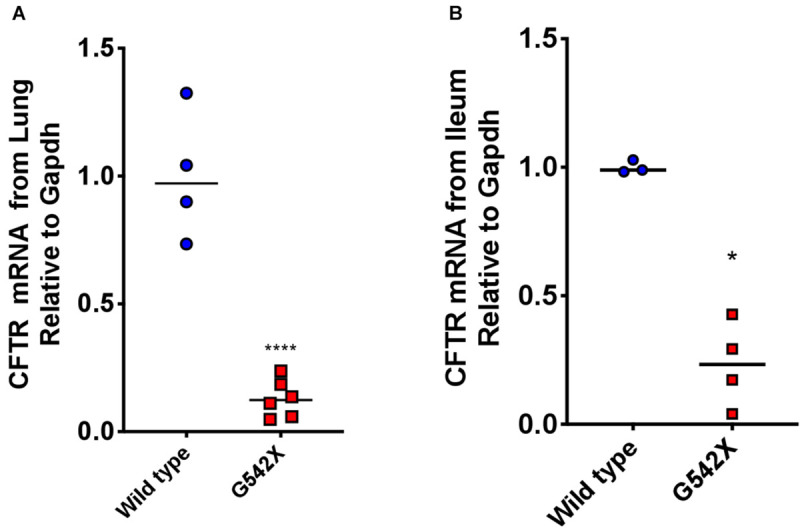
Rescued CFTR transcript levels from lungs and ileal sections of G542X CFTR rats. CFTR transcript levels were evaluated using qRTPCR. **(A)** CFTR mRNA levels relative to Gapdh from left lung section from G542X and wild type CFTR rats. **(B)** CFTR mRNA levels relative to Gapdh from ileal sections. *N* = 3–6 animals/group. ^****^P < 0.0001 by ANOVA with Tukey *post hoc* testing.

Nonsense-mediated decay plays an important role in genetic diseases caused by nonsense mutations by degrading PTC-bearing transcripts and can limit therapeutic efficiency of readthrough agents (Linde et al., 2007; Silva and Romao, 2009; Kervestin and Jacobson, 2012; Keeling et al., 2013). This provided a major impetus for the development of a knockin G542X CFTR rat, rather than a transgenic species not subject to NMD. To determine the degree of NMD-mediated degradation of CFTR mRNA, we measured CFTR transcript levels in G542X and wild type rats. Considering that NMD intensity can exhibit different levels in different tissues (Geiger et al., 2008; Zetoune et al., 2008; Thada et al., 2016), we examined mRNA levels in both the right lung and ileum. Steady-state CFTR mRNA levels relative to Gapdh were 6 times less abundant in G542X rat lung and ileal sections compared to wild type (16% of wild type, *P* < 0.0001 and *P* < 0.05, Figures 5A,B, respectively), indicating the presence of robust G542X mRNA degradation. Comparison with additional reference genes (Rps6 and Hprt) showed similar findings (Supplementary Figures 2A,B).

### Characterization of RTECs

**FIGURE 6:**
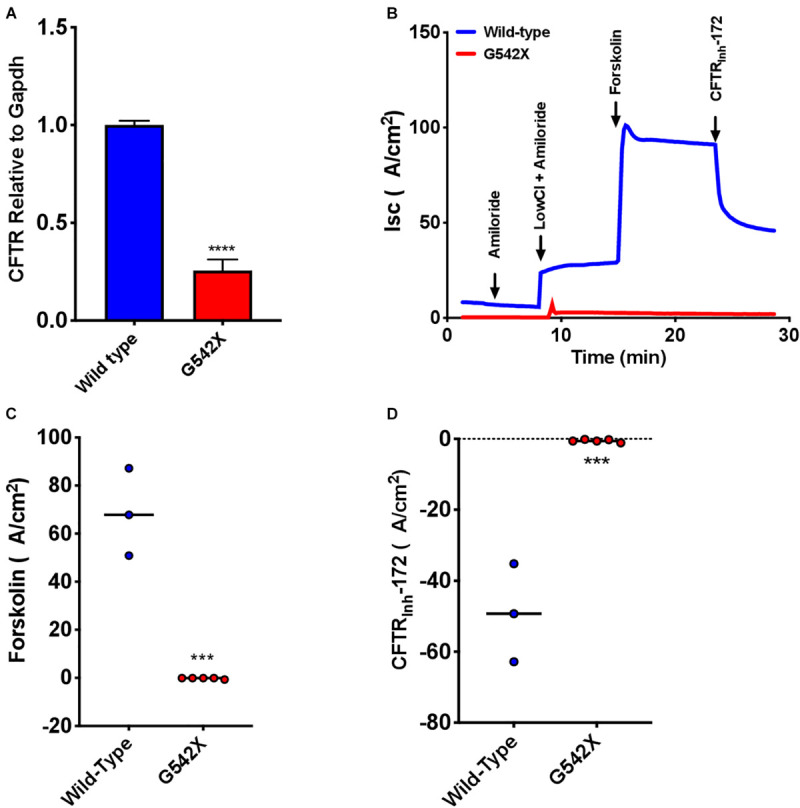
Absent CFTR mRNA expression and CFTR-dependent I_sc_ in rat tracheal epithelial cells (RTECs) derived from G542X and wild type rats. Cells were grown on transwell filters until terminal differentiation. **(A)** CFTR mRNA levels relative to Gapdh in G542X cells compared to wild type RTECs. **(B)** Representative I_sc_ tracings in RTECs from each group showing serial addition of amiloride, low Cl + amiloride, forskolin, and CFTR_inh_-172. **(C,D)** Summary data showing change in forskolin-stimulated I_sc_ **(B)** and CFTR_inh_-172 mediated inhibition **(C)**. *N* = 3–6 monolayers/condition. ^∗∗∗^*P* < 0.001, ^****^P < 0.0001 by ANOVA with Tukey *post hoc* testing.

To examine treatment modalities, address the poor availability of human bronchial epithelial cells from donors homozygous for the same PTC, and maximally utilize the G542X rat model, we next characterized primary RTECs cultured at air-liquid interface. We first examined CFTR transcript levels obtained from RTECS derived from G542X and wild type rats, and found a 4-fold mRNA reduction in G542X animals (*P* < 0.0001, Figure 6A). This suggested that G542X mRNA was subjected to NMD degradation in cell culture of isolated epithelial cells, as in tissues. We then evaluated CFTR-associated I_sc_. CFTR G542X RTECs showed significantly reduced forskolin-stimulated I_sc_ compared to wild type RTECs (G542 X = -0.3 ± 0.2 μA/cm^2^, wild type = 68.6 ± 10.5 μA/cm^2^, *P* < 0.001, Figures 6B,C). In addition, I_sc_ was reduced by addition of CFTR_Inh_-172 in wild type, but not G542X RTECs (G542X = -0.3 ± 0.2 μA/cm^2^, wild type = -49.1 ± 8.0 μA/cm^2^, *P* < 0.001, Figures 6B–D). These findings further support the absence of CFTR activity in CFTR G542X knockin rats.

### Treatment With Readthrough Agents Rescues CFTR Function *in vitro*

To examine CFTR functional improvements in response to readthrough treatment therapies, we next evaluated the aminoglycosides G418 and amikacin, agents known to induce translational readthrough (Manuvakhova et al., 2000; Du et al., 2006), in G542X RTECs. Dose-response studies evaluating G418 at a dose range of 1–100 μM and amikacin at a range of 50–341.5 μM revealed that doses greater than 12.5 μM (G418) and 341.5 μM (amikacin) disrupted the cell monolayer and corresponding I_sc_ tracings (Supplementary Figures 3A–D). At the optimal dose, G418 (3 μM) elicited statistically significant rescue of CFTR function (1.4 ± 0.6 μA/cm^2^, *P* = 0.05), but only approached ∼3% of wild type RTEC CFTR activity (68.63 ± 18.2 μA/cm^2)^, while amikacin (170.8 μM) did not improve function (Figures 6A,B). The low efficacy of these aminoglycosides in rescuing CFTR function is likely due in part to the significantly reduced transcript levels (*P* < 0.05, Figures 5A,B) for which single readthrough agent treatment may not be sufficiently efficient in overcoming.

### Readthrough Treatment Response *in vivo*

Although amikacin was not active *in vitro*, we next evaluated the activity of amikacin *in vivo* based on the finding that amikacin is more efficacious than gentamicin in humanized CFTR G542X transgenic mice (McHugh et al., 2018) and noting that G418 is toxic *in vivo*. To accomplish this, amikacin was administered to G542X rats via subcutaneous injection at a high dose (170 mg/kg daily for 12 days). The steady-state plasma peak and trough levels of amikacin were 177.5 ± 37.5 μg/ml and 0.6 ± 0.2 μg/ml, respectively, 24 h after the last treatment, achieving adequate levels. We did not observe any adverse effects in the treated rats, as determined by body weight, measured daily during the treatments. To determine the nonsense suppression effect of amikacin, we then measured I_sc_ in tracheal and intestinal sections following the 12-day treatment. While baseline tracheal currents were significantly higher in amikacin-treated G542X rats (treated −206.5 ± 35.2 μA/cm^2^ vs. untreated −25.5 ± 9.1 μA/cm^2^, *P* < 0.05, Figures 8A,B), a potential indicator of a treatment effect, I_sc_ reduction with CFTR_Inh_-172 was no greater in amikacin-treated (2.9 ± 5.5 μA/cm^2^) vs. untreated (−7.3 ± 4.4 μA/cm^2^) rats (Figures 8A,C), indicating this effect was likely not specific to CFTR. We conclude that although amikacin as a readthrough agent treatment was well-tolerated, there was no significant treatment response likely because aminoglycoside therapy could not overcome the effect of reduced levels of CFTR transcripts via NMD.

## Discussion

In this study, we have generated and characterized a novel CF rat model for the CFTR nonsense mutation G542X. To the best of our knowledge, this is the first report of a rat model expressing a CFTR nonsense mutation. This model will offer new opportunities to investigate the ramifications of CF disease progression, severity, and respiratory pathology resulting from CFTR nonsense mutations in a longitudinal manner (Birket et al., 2018), as well as provide a relevant animal model to study emerging therapies where the mucus defect (Birket et al., 2018) or the pharmacology of CFTR (Birket et al., 2016) are important parameters. As CFTR nonsense mutations remain the largest untreated mutation type among CF patients, this should help expedite research into these efforts, as well as other diseases for which nonsense mutations play an important role.

CFTR G542X knockin rats demonstrated severe CF manifestations and developmental defects. CF-related abnormalities included reduced growth, aberrant tooth enamel, and meconium ileus (Figure 1), similar to observations in CF rodent models and patients (Wright et al., 1996; Arquitt et al., 2002; Meyerholz et al., 2010; Tuggle et al., 2014; Stalvey et al., 2017). Reduced CFTR expression and activity was evident in multiple organs (Figures 2–4). As seen in adult CF knockout rats (Tuggle et al., 2014), G452X knockin rats exhibit the unique electrophysiological phenotype with constitutively active baseline CFTR current in *ex vivo* tracheal sections, prior to cAMP stimulation (Figure 3 and Supplementary Figure 1), as opposed to the neonatal rats where pre-activation is not apparent (McCormick et al., 2018). NPD results (Figure 2) were similar to knockout rats of similar age (Tuggle et al., 2014).

Importantly, G542X knockin rats exhibited sensitivity to NMD (Figure 5), a pathway highly relevant to the rescue of nonsense mutations but distinct from transgenic mouse models which do not exhibit this property (Du et al., 2006). CFTR transgenic mice express human CFTR cDNA containing the *G542X* mutation, but this does not result in intron splicing that triggers NMD when a PTC is encountered during the pioneer round of translation (Maquat, 2004; Du et al., 2006); this substantially reduces the predictive capacity of transgenic mice since humans with CFTR nonsense mutations exhibit NMD sufficient to reduce transcript levels to 20–40% of normal (Sharma et al., 2018; Clarke et al., 2019) and NMD is also known to alter drug response to pharmacological therapy directed against nonsense mutations (Linde and Kerem, 2011; Sharma et al., 2020). Further, the human CFTR cDNA in G542X mice is driven by a rat Fatty Acid Binding Protein (FABP) promoter that results in high levels of intestinal-specific expression, obviating the effects of low transcript levels and limiting tissue assessments to the intestine where it is expressed (Du et al., 2006). CFTR G542X rats should provide a much more relevant system in these respects, while also providing organ systems suitable for monitoring biochemical and functional rescue of CFTR, in addition to gastrointestinal and respiratory phenotypes. Future studies can be implemented to understand the complexity of the specific NMD branches involved in the recognition and degradation of the G542X CFTR mutation, potentially leading to new insights and therapeutic opportunities, since inhibition of NMD is under consideration as a therapeutic approach (Huang et al., 2018; Sharma et al., 2020). The G542X rat can also be used to evaluate drugs in development targeting PTCs, including those identified from high-throughput screens.

**FIGURE 7:**
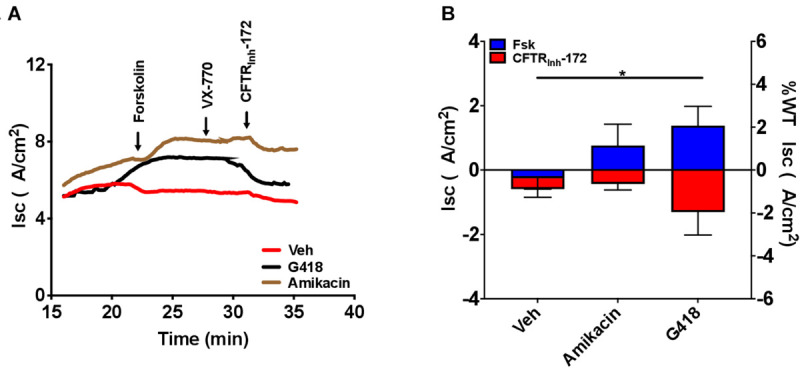
Increased CFTR-dependent I_sc_ with treatment with the translational readthrough agent G418 but not amikacin in CFTR G542X RTECs. RTECs were grown until terminally differentiated and then treated with the readthrough agents G418 (3 μM) and amikacin (170.8 μM) for 48 h before the assay. **(A)** Representative I_sc_ tracings for each condition showing serial perfusion with forskolin (FSK), VX-770, and CFTR_inh_-172 (10 μM, each). **(B)** Summary data of forskolin-stimulated and CFTR_inh_-172-inhibited I_sc_. *N* = 2–5 filters/condition, ^∗^*P* < 0.05 by ANOVA with Tukey *post hoc* testing.

**FIGURE 8:**
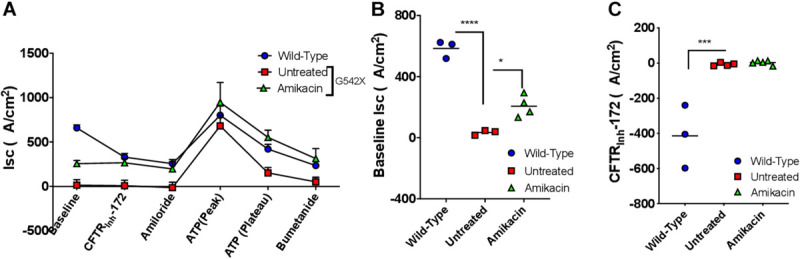
The effect of amikacin treatment *in vivo* on effects on tracheal currents in G542X rats. Rats were treated with amikacin (170 mg/kg) subcutaneously daily for 12 days, then underwent necropsy for tissue analysis. **(A)** I_sc_ summary tracings. **(B)** Baseline I_sc_ in G542X (treated and untreated) and wild type animals. **(C)** CFTR_Inh_-172-mediated inhibition in G542X (treated and untreated) and wild type rats. *N* = 3 animals/group. ^∗^*P* < 0.05, ^∗∗∗^*P* < 0.001, ^****^P < 0.0001 by ANOVA with Tukey *post hoc* testing.

To help establish the principle that G542X CFTR knockin rats could be useful in pharmacological drug development for nonsense mutations, we evaluated translational readthrough induced by clinically available aminoglycosides in addition to the more efficacious tool compound G418 (geneticin) *in vitro*. We chose aminoglycosides since they are the best studied among readthrough agents, but note that aminoglycoside-mediated translational readthrough has shown mixed results in published studies since efficacy is marginal. Aminoglycosides have partially restored CFTR expression and/or function *in vitro* and *in vivo* in some studies (Howard et al., 1996; Bedwell et al., 1997; Barton-Davis et al., 1999), while in several others no definitive rescue of full-length functional protein has been shown (Wilschanski et al., 2000; Dunant et al., 2003; Politano et al., 2003; Howard et al., 2004). Unfortunately, while *in vitro* activity could be observed with G418 in RTECs (Figure 7), the safer but less active aminoglycoside amikacin was not sufficient to restore CFTR activity either *in vitro* or *in vivo* (Figure 8). While there was small degree of normalization of baseline I_sc_ of excised trachea, this was not accompanied by increased forskolin stimulated I_sc_, nor correction of NPD; we interpret this as an effect not specific to induction of translational readthrough. This differs from findings in transgenic G542X expressing mice, where amikacin (Du et al., 2006), in addition to gentamicin (Du et al., 2002) and other readthrough agents (Rowe et al., 2011; Xue et al., 2014), have been shown to exhibit bioactivity. We expect this is related to preserved CFTR mRNA in mice, but not in knockin rats *in vivo*. The mRNA degradation in G542X knockin rats was sufficient to comprise detection of the low-level readthrough induced by amikacin (Figures 5,6), but does not impact the G542X transgenic models that are not affected by reduced mRNA transcript levels. It may be why some therapies have shown to be efficacious in transgenic G542X mice, but not active in human subjects (Clancy et al., 2007). We suspect the G542X rat will provide a more specific test of this question, and can be implemented sequentially with transgenic mice within a drug evaluation program for that purpose.

Supporting the use of G542X rats for *in vivo* applications, we also developed a method to procure and test RTECs. This may help address the limited availability of primary human bronchial epithelial cells from individuals homozygous for CFTR nonsense mutations that are necessary to assess readthrough molecules and the concomitant use of CFTR modulators. RTECs provide a potentially unlimited source to explore novel therapies for nonsense mutations, and are amenable to I_sc_ analyses that are a mainstay of CFTR modulator development. The close relationship between the properties of RTECs and the *in vivo* and *ex vivo* evaluation of G542X rats with respect to biochemical CFTR expression and electrophysiological CFTR function suggest they will predict *in vivo* response to rats, complementing reporter assays or transfected cell lines. Ultimately, given RTECs are primary in nature, and are not devolved by repeated passaging, these cells may better reflect the *in vivo* context and might ultimately be used to help predict efficacy, as primary human bronchial epithelial cells have been implemented for CFTR modulator therapies (Clancy et al., 2019). It should be noted that the CFTR sequence is native to rats, so treatments dependent on the human sequence may not be reflected in studies involving the G542X knockin rat.

Our results with amikacin treatments *in vitro* or *in vivo* demonstrated that single readthrough agent therapy is not sufficient to surpass the therapeutic threshold for CF nonsense mutations when not over-expressed, and evaluated in the absence of an active CFTR modulator to augment post-translational CFTR activity of the resulting protein. The sensitivity to NMD can cause serious repercussions for these therapies, including dampening the efficacy of readthrough by lowering the substrates available for readthrough treatment. Inhibition of NMD could be a potential way to improve the availability of PTC-containing transcripts for readthrough therapy. In addition, approaches like tRNA suppression or gene therapies might prove beneficial for CF patients bearing nonsense mutations and this knockin G542X rat model provides an important tool to test these therapies. Multi-agent therapy to augment readthrough could be an alternative approach, and ultimately evaluated in *in vivo* models such as the G542X rat we have developed here.

## Data Availability Statement

The original contributions presented in the study are included in the article/Supplementary Material, further inquiries can be directed to the corresponding author/s.

## Ethics Statement

The animal study was reviewed and approved by University of Alabama at Birmingham (UAB) Institutional Animal Care and Use Committee 94 (IACUC; UAB Approval Number 09479).

## Author Contributions

SR provided reagents and technique. SR, LK, JA, GZ, and SB helped conceive of the genetic engineering of the model. SB maintained rat colonies and provided rats. JS, JA, LK, and GZ conducted the research. JS and SR analyzed the data and wrote the manuscript. SB and SR supervised the project. All authors had an opportunity to edit the manuscript and approved of its submission.

## Conflict of Interest

SR receives grant funding and consulting fees from pharmaceutical companies developing CF therapies that could benefit from use of the G542X rat model, including translational readthrough agents and genetic therapies; a complete list of all potential conflicts has been included in the ICJME form. JA, LK, and GZ are employed by the company Horizon Discovery Group. The remaining authors declare that the research was conducted in the absence of any commercial or financial relationships that could be construed as a potential conflict of interest.
