# Supplementary material for: A Novel G542X CFTR Rat Model of Cystic Fibrosis Is Sensitive to Nonsense Mediated Decay
Source: Front Physiol. 2020 Dec 16;11:611294. doi: 10.3389/fphys.2020.611294 (PMC7772197; doi:10.3389/fphys.2020.611294)
Supplement: Supplementary file 1 [file Table_1.DOCX]

Supplementary Material

**Representative WT Sequence-**

CCGGACCCGAGCATGCACTATCGCCTCGGTTGAATAGTTGATAGAGGAATCTGACATACAGTTGCATCTGAAGAAAAATG

TCCTTTTTAATTTCAGATTGCATATACTAAATTTTATTTCTGGTGTTATGCTTTGAATAATAGGACATCACCAAGTTTGC

AGAACAAGACAACACAGTTCTTGGAGAAGGTGGAGTCACACTCAGTGGAGGTCAACGTGCAAGAATTTCTTTAGCAAGGT

AAACGTTCAACTGTTGGTTTGCTGAGAACTTGCTGTAAATGGATGTTTTATAATATACACACTTCTCTTCTGCTTGTGCT

CTGTCTCTAGGGA

**Representative G542X Sequence-** CCGTACCTAGCATGCAGTATGCGTCGATTGAATAGTTGATAGAGGAATCTGACATACAGTTGCATCTGAAGAAAAATGTC

CTTTTTAATTTCAGATTGCATATACTAAATTTTATTTCTGGTGTTATGCTTTGAATAATAGGACATCACCAAGTTTGCAG

AACAAGACAACACAGTTCTTTGAGAAGGTGGAGTCACACTCAGTGGAGGTCAACGTGCAAGAATTTCTTTAGCAAGGTAA

ACGTTCAACTGTTGGTTTGCTGAGAACTTGCTGTAAATGGATGTTTTATAATATACACACTTCTCTTCTGCTTGTGCTCT

GTCTCTAGGGA

**Supplementary Figure 1. Functional defects in the lung of G542X CFTR rats.**

Representative tracheal I_sc_ tracing from G542X and WT rats with perfusion of ringers, amiloride (100 µM), forskolin (10 µM), ATP (10 µM) and bumetanide (100 µM).

**Supplementary Figure 2- CFTR Transcript levels in G542X and WT rats.** (A) CFTR mRNA levels relative to RPS9 by RT-PCR. (B) CFTR mRNA levels relative to HPRT.

**Supplementary Figure 3. Short circuit currents and TEER measurements in CFTR G542X RTECs after treatments with amikacin and G418.** RTECs were grown on ALI culture until terminally differentiated then cells were treated with readthrough agents for 48 hr before the assay (A) Representative I_sc_ tracings following G418 treatment (1.5 to 25 µM). (B) Representative I_sc_ tracings for Amikacin (85.4, 170.8 and to 341.5 µM). TEER (C) G418, 1.5 to 100 µM. (D) Amikacin, 85.4, 170.8 and 341.5 µM.
